# The essential role of glutamine metabolism in diabetic cardiomyopathy: A review

**DOI:** 10.1097/MD.0000000000036299

**Published:** 2023-11-24

**Authors:** Yiying Zhang

**Affiliations:** a Department of Cardiovascular Medicine, Wuxi No.2 People’s Hospital, Wuxi City, People’s Republic of China.

**Keywords:** diabetic cardiomyopathy, ferroptosis, glutamine metabolism, inflammation, O-GlcNacyation

## Abstract

Diabetic cardiomyopathy (DCM) is a pathophysiological condition caused by diabetes mellitus and is the leading cause of diabetes mellitus-related mortality. The pathophysiology of DCM involves various processes, such as oxidative stress, inflammation, ferroptosis, and abnormal protein modification. New evidence indicates that dysfunction of glutamine (Gln) metabolism contributes to the pathogenesis of DCM by regulating these pathophysiological mechanisms. Gln is a conditionally essential amino acid in the human body, playing a vital role in maintaining cell function. Although the precise molecular mechanisms of Gln in DCM have yet to be fully elucidated, recent studies have shown that supplementing with Gln improves cardiac function in diabetic hearts. However, excessive Gln may worsen myocardial injury in DCM by generating a large amount of glutamates or increasing O-GlcNacylation. To highlight the potential therapeutic method targeting Gln metabolism and its downstream pathophysiological mechanisms, this article aims to review the regulatory function of Gln in the pathophysiological mechanisms of DCM.

## 1. Introduction

Diabetes mellitus (DM) is the most common metabolic illness characterized by hyperglycemia and is currently a significant public health burden worldwide. In 2017, the global prevalence of DM was 476 million, projected to increase to 570.9 million in 2025.^[1]^ Diabetic cardiomyopathy (DCM) is a major contributor to the mortality associated with diabetes and is a non-ischemic, non-hypertensive pathophysiological state induced by the disease. Patients with DCM often exhibit impaired diastolic dysfunction and left ventricular hypertrophy, while systolic function is typically normal in the early stage and systolic dysfunction occurs in the middle and late stages.^[2,3]^

Although significant advancements have been made in investigating DCM in the past few decades, the pathogenesis of DCM remains unclear. Multiple mechanisms are thought to be involved in the pathogenesis of DCM, including oxidative stress, inflammation, mitochondrial dysfunction, cardiomyocyte apoptosis, necrosis or ferroptosis, autophagy, endoplasmic reticulum stress, and dysfunction of amino acid metabolism, especially glutamine (Gln) metabolism.^[4]^ Among these pathophysiological mechanisms, Gln metabolism dysfunction plays a fundamental role in the pathology of cardiomyopathy and has been found to be a critical factor in the pathogenesis of DCM.^[5]^

Gln, classified as a conditionally essential amino acid, is the most abundant amino acid in the human body. Emerging evidence suggests that it plays an essential role in regulating cell function by serving as a crucial substrate in many biosynthetic pathways, such as peptide and protein synthesis, lipid metabolism, the synthesis of nitrogenous bases, and the production of antioxidants and nicotinamide adenine dinucleotide phosphate.^[6]^ Gln is primarily synthesized from L-glutamate and ammonia by Gln synthetases, which are mostly enriched in the cytoplasm. Simultaneously, Gln is hydrolyzed to glutamate (Glu) and NH3 by two different glutaminase (GLS) isoforms, GLS1 and GLS2. GLS is an enzyme found in the mitochondria, and GLS1 is mainly responsible for Gln hydrolysis in cardiovascular tissues.

**Figure 1.**
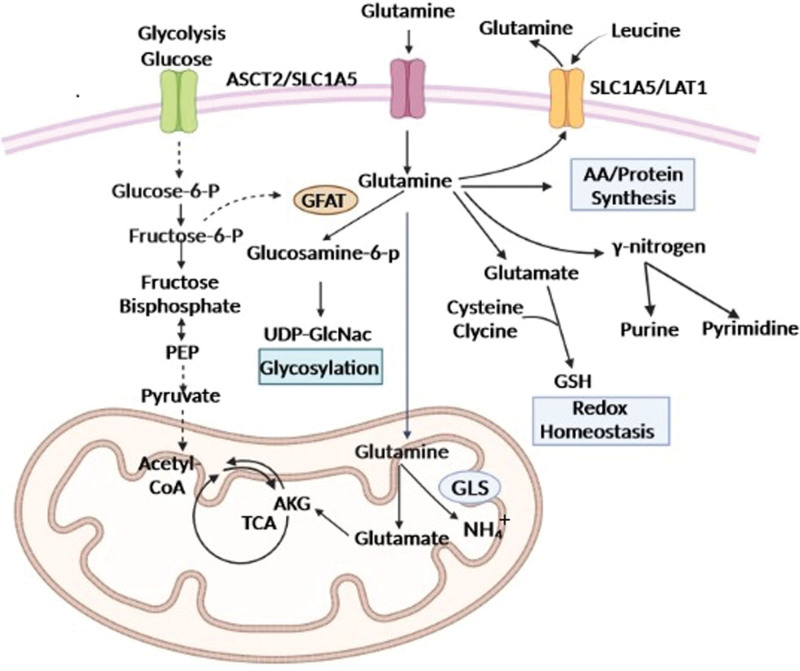
Gln metabolism pathway.

As shown in Figure 1, extracellular Gln is transported into cells via two amino acid transporters, System adaptor protein caspase-1 recruitment domain amino acid transporter-2 and Solute Carrier Family 1 Member 5 (SLC1A5), and is predominantly catabolized by GLS in the mitochondria into Glu. Glu can then be converted into alpha-ketoglutarate (AKG) by Glu dehydrogenase in the mitochondria, or by aminotransferases in either the cytosol or the mitochondria. AKG can be used to generate adenosine triphosphate (ATP) via the tricarboxylic acid cycle or can be utilized for the synthesis of non-essential amino acids and lipids. Under conditions of hypoxia or mitochondrial dysfunction, AKG can produce nicotinamide adenine dinucleotide phosphate, which plays a crucial role in redox homeostasis through malate-pyruvate cycling, and additionally produces amino acids and lipids.^[7,8]^ In the cytosol, some Gln is also metabolized by providing γ-nitrogen for the synthesis of nucleotides and hexosamine, and during this process, Glu is produced. Cytosolic Glu is then used to synthesize the antioxidant glutathione, which protects cells from oxidative stress. Furthermore, Gln can also be metabolized by Gln fructose-6-phosphate aminotransferase, a key enzyme of the hexosamine pathway, which converts Gln and fructose-6-phosphate to glucosamine-6-phosphate, an essential precursor for posttranslational protein glycosylation.^[9]^

The importance of Gln in nutrition and health has been well established, and in recent years, its role in protecting the heart has become increasingly apparent.^[10–14]^ Furthermore, a growing body of research has shed light on the intricate function of Gln metabolism in the pathogenesis and progression of DCM.^[15–18]^ In this review, we briefly summarize the function of Gln metabolism in the pathology of DCM and highlight potential therapeutic approaches that target this amino acid in DCM.

## 2. The role of Gln in DCM

The function of Gln in diabetes and cardiomyopathy has been extensively studied. It has been reported that serum Gln is associated with a lower risk of type 2 diabetes (T2D), while Glu is associated with a higher risk of T2D.^[19]^ Additionally, patients with diabetes exhibit alterations in Gln metabolism, as evidenced by lower serum concentrations of Gln and AKG.^[20]^ Diabetes is characterized by inflammation, and macrophages have been shown to be responsible for the inflammation in diabetes. M1 macrophages are associated with the progression of diabetes, while macrophage polarization from M1 to M2 may have a potential therapeutic effect on diabetes. Several studies have indicated that Gln in serum is essential for the polarization of M2 macrophages by producing AKG through glutaminolysis and acting as a metabolic substrate for the hexosamine pathway.^[20]^ In type 1 diabetes (T1D), Gln is helpful for glycemic control, as oral supplementation of Gln can decrease blood glucose in adolescents with T1D.^[21]^ Furthermore, in mice with streptozotocin-induced T1D, dietary Gln can increase antioxidant potential and decrease the expression of leukocyte adhesion molecules in the liver and/or kidney.^[22]^

The protective role of Gln in heart health has been clearly demonstrated. Inhibiting cellular Gln uptake can limit the production of cellular energy of cardiomyocytes,^[23]^ while supplementing with Gln can promote oxidative phosphorylation of cardiomyocytes, reducing myocardial injury and clinical complications.^[24,25]^ With the elucidation of the cardioprotective effect of Gln, its protective effect on DCM has also attracted attention.

Recent studies have suggested that Gln plays a protective role in DCM, mainly through anti-oxidative stress, maintenance of the heart structure, or modulation of protein O-GlcNacylation (O-GlcNAc). Gln supplementation can dramatically increase the levels of endogenous antioxidant enzymes (superoxide dismutase, glutathione (GSH), and malondialdehyde) in the heart of diabetic rats, preventing the development of experimental DCM.^[15]^ Gln treatment in vivo can also protect cardiomyocytes from hypoxia/reoxygenation injury under high glucose conditions by inhibiting cell apoptosis.^[26]^ Gln supplementation can enhance the expression of Heat shock proteins 70 in diabetic rats, exerting a cardioprotective effect in left ventricular tissues.^[16]^ As an important substrate of the hexosamine pathway, Gln can be converted to the precursors for the posttranslational protein glycosylation, O-GlcNAc, which has been shown to promote the progression of DCM when overexpressed.^[18]^ These findings suggest that Gln supplementation has a compensatory protective effect on DCM using different approaches and different in vitro and animal models of T2D and/or T1D, but the underlying mechanism requires further elaboration.

## 3. Mechanisms underlying the protective role of Gln in DCM

DCM is a multifactorial condition that involves various pathological mechanisms at the systemic, cardiac, and cellular/molecular levels, including oxidative stress, inflammation, protein modification, mitochondrial dysfunction, inadequate energy supply, cardiac remodeling, apoptosis, and ferroptosis.^[3]^ Among these mechanisms, oxidative stress, inflammation, protein modification, and ferroptosis are closely related to Gln metabolism. The role of Gln in these mechanisms is elaborated below.

## 4. Oxidative stress

Oxidative stress is characterized by an imbalance between the production of reactive oxygen species (ROS) and antioxidant defenses.^[27]^ ROS overproduction damages cell membranes and essential biomolecules, including lipids, proteins, and DNA, and is associated with reduced insulin secretion and insulin resistance.^[27]^ In T1D, oxidative stress plays a crucial role in the destruction of insulin beta cells due to inflammatory cytokines and autoimmune responses.^[28]^ The pivotal role of oxidative stress in the pathogenesis of DCM has been demonstrated in numerous studies. Excessive ROS in cardiomyocytes leads to further ROS production, resulting in mitochondrial dysfunction, excessive autophagy, lipid peroxidation, abnormal protein post-translational modifications, and ultimately cell death and cardiac dysfunction.^[29–32]^

Recent studies have highlighted the role of Gln in protecting against oxidative stress in DCM. Morotti et al^[33]^ reported that upregulation of Gln transporter sodium-dependent neutral amino acid transporter-2/solute carrier family 38 member promotes oxidative stress resistance. GSH, an important oxidant produced by glutaminolysis, is depleted during oxidative stress. Upregulation of glutaminolysis under oxidative stress conditions can supplement AKG to the tricarboxylic acid cycle to maintain ATP and GSH production, thereby exerting cardioprotective effects. Inhibition of GLS reduces ATP and GSH synthesis, further decreasing cell viability.^[34]^ In addition, Gln can enhance the activity of antioxidant enzymes such as superoxide dismutase and GSH-peroxidase by activating the wingless-type MMTV integration site family, member 3A/β-Catenin signaling pathway.^[35]^ Activation of the phosphatidylinositol 3-kinase (PI3K)/PKB (protein kinase B)–endothelial nitric oxide synthase pathway also contributes to the amelioration of oxidative stress damage.^[36]^ Suppression of the PI3K/PKB signaling pathway has been associated with oxidative stress and cardiac dysfunction in DCM, while activation of the PI3K/PKB pathway has been found to improve left ventricular dysfunction, cardiac hypertrophy, myocardial interstitial fibrosis, and remodeling in DCM mice.^[37]^ Another recent study showed that Gln could activate the PI3K/PKB signaling pathway and protect against myocardial ischemia-reperfusion injury,^[10]^ suggesting that Gln can inhibit oxidative stress through activating the PI3K/PKB signaling pathway in DCM. Previous studies have demonstrated that L-Gln supplementation can reduce oxidative stress parameters and improve cardiac function in diabetic rats.^[15]^ Additionally, Erzhi Pill, a traditional Chinese formulation, has been shown to improve oxidative stress in DCM, probably by modulating Gln metabolism.^[38]^ Taken together, these studies suggest that Gln can ameliorate oxidative stress injury and thereby improve DCM.

## 5. Inflammation

Inflammation is a hallmark of diabetes, and it plays a significant role in the pathology of DCM. High glucose levels trigger an inflammatory reaction that leads to cardiomyocyte apoptosis.^[39]^ Elevated levels of inflammation markers, including interleukin-6 (IL-6), tumor necrosis factor α (TNF-α), and C-reactive protein, have been associated with worsening cardiovascular disease.^[40–42]^ Furthermore, activation of NF-κB (nuclear factor-κB) is commonly observed, leading to increased production of proinflammatory cytokines, ultimately contributing to DCM.^[43]^ However, inhibiting the NF-κB signaling pathway can protect cardiomyocytes from apoptosis caused by high glucose levels.^[43,44]^ Toll-like receptor 4 (TLR4), a crucial element of the innate immune system, plays a substantial role in the progression of DCM. It accomplishes this by triggering proinflammatory pathways in response to elevated glucose levels through the recruitment of two vital adaptor molecules: myeloid differentiation primary response-88 (MyD88) and TIR-domain-containing adapter-inducing interferon-β, ultimately leading to the activation of NF-κB.^[45]^ Increased TLR4 expression has been observed in the heart tissues of diabetic mice and rats.^[46,47]^ The inhibition of the TLR4-induced NF-κB/IL-1β immune pathway has demonstrated a protective effect against the development of DCM.^[47]^ Additionally, interventions targeting the interaction between MyD88 and TLR4 or inhibiting MyD88 itself have shown promise in safeguarding cardiac tissues in both type 1 and type 2 diabetic murine models, effectively preventing the activation of the MyD88-dependent proinflammatory signaling pathway.^[48]^

The activation of nucleotide-binding domain, leucine-rich-containing family, pyrin domain-containing-3 inflammasome plays a crucial role in the development of DCM, as evidenced by previous studies.^[49–54]^ These studies have demonstrated that hyperglycemia can activate NLRP3, subsequently activating caspase-1. This activation facilitates the maturation of pro-IL-1β and the activation of Gasdermin-D, ultimately leading to the formation of membrane pores and triggering inflammation-related programmed cell death, which called pyroptosis.^[49–53]^ Recent studies have shown that inflammation is involved in pyroptotic cell death and cardiac remodeling in diabetic hearts.^[55]^ The suppression of the NLRP3 inflammasome has been shown to ameliorate cardiac function by reducing cardiac inflammation, pyroptosis, and fibrosis in DCM mice and rats, providing substantial protection against DCM.^[56–58]^ Furthermore, C-Jun N-terminal kinase (JNK) also contributes to the pathogenesis of DCM, and inhibition of its phosphorylation in T1DM mice has been shown to significantly reduce plasma and cardiac pro-inflammatory cytokines, improving cell viability and cardiac function without affecting hyperglycemia.^[39]^

Despite the activation of inflammatory signaling pathways, the activation of certain immune cells plays a crucial role in inducing inflammation in DCM.^[59,60]^ Under high glucose conditions, macrophages assume an M1 pro-inflammatory phenotype, secreting TNF-α, IL-6, and C-C motif ligand 2. However, inhibiting macrophage polarization towards M1 phenotypes and promoting the polarization of M2 macrophages can reduce the inflammatory response and improve cardiac fibrosis in DCM.^[61]^ T lymphocytes have also emerged as key players in the pathogenesis of diabetic cardiomyopathy. Elevated T cell counts in diabetic patients are associated with a higher risk of diabetic cardiovascular complications.^[62,63]^ Infiltration of T lymphocytes has been observed in the cardiac tissues of diabetic mice, and the depletion of T cells has been shown to significantly improve cardiac fibrosis and myocardial dysfunction.^[64]^ Furthermore, regulatory T (Treg) cell subsets, known for their immunosuppressive properties, have a protective role in cardiovascular diseases by responding to inflammatory signals.^[65]^ Suppressing Treg cells can promote cardiac fibrosis in DCM mice, while restoring the population of circulating Treg cells significantly improves cardiac function in mice with DCM.^[66,67]^

Numerous studies have reported that supplementing with Gln can significantly decrease the levels of pro-inflammatory cytokines, such as IL-6, TNF-α, interleukin-1β (L-1β), and C-reactive protein, in multiple diseases.^[68–70]^ The administration of Gln suppresses the activation of NF-κB and promotes the degradation of IκBα by enhancing cullin1 deneddylation.^[71]^ Lesueur et al^[72]^ demonstrated that Gln can also inhibit the NF-κB pathway by inducing the degradation of nuclear p65 subunits. Glutamine supplementation alleviates lipopolysaccharide-induced inflammatory responses by inactivating various signaling pathways, including TLR4/focal adhesion kinase/MyD88, TLR4/NF-κB, and TLR4/mitogen-activated protein kinases pathways.^[73–76]^ In a double-blind, randomized, controlled pilot trial involving patients diagnosed with pelvic or abdominal malignancies undergoing abdominal radiotherapy, oral glutamine supplementation significantly reduced the expression of TLR-4 and NF-κB.^[77]^ However, it’s important to note that there have been conflicting findings in the literature. For instance, Sukhotnik et al^[78]^ reported that the expression of TLR4 and MyD88 was significantly decreased in methotrexate-induced mucositis rats, whereas treatment with glutamine resulted in the up-regulation of TLR-4 and MyD88. Moreover, in patients admitted to the ICU who received parenteral nutrition supplemented with glutamine, there was no observed effect on the expression of TLR-4 in peripheral blood monocytes.^[79]^ These contradictory results suggest that the role of glutamine in TLR4 expression and the activation of downstream signals may vary under different physiological conditions. Furthermore, glutamine administration has been shown to suppress inflammation by inhibiting the activation of NLRP3 and modulating cell pyroptosis.^[80,81]^ Zhang et al^[82]^ demonstrated that inhibition of glutaminolysis can inhibit the activation of the NLRP3/caspase-1/IL-1β pathways. The activation of JNK is dependent on the phosphorylation of JNK, while treatment with Gln can decrease the phosphorylated JNK by attenuating inflammation.^[75,83]^

AKG, produced through AKG glutaminolysis, can promote the polarization and activation of M2 macrophages. Inhibition of GLS1 can significantly decrease the infiltration and M2 polarization of macrophages.^[84]^ In terms of mechanism, AKG mainly modulates the polarization of M2 by increasing fatty acid oxidation and oxidative phosphorylation in M2 macrophages. In additon, a recent study showed that AKG can also induce the M2 phenotype through activating Jumonji domain-containing protein-3, which can demethylate histone H3 lysine 27 in the promoter region of M2-specific marker genes.^[85]^

Glutamine metabolism has also been reported to play a critical role in modulating T cell activation. Suppression of GLS can significantly enhance T cell activation.^[86]^ In the context of lung injury induced by polymicrobial sepsis, glutamine administration was found to alleviate inflammation by reducing blood helper T cell 17, helper T cell 1, and helper T cell 2 cells while promoting Treg cell populations.^[87]^ Additionally, GSH has demonstrated a protective effect on preserving Treg functionality.^[88]^ These studies collectively indicate that glutamine can modulate inflammation by affecting T cell activation. However, it’s worth noting that some studies have presented an opposing viewpoint. It has been suggested that excessive glutamine consumption may contribute to T cell activation.^[89]^ Moreover, inflammatory T cell responses have been associated with increased glutamine uptake, and blocking amino acid transporters such as adaptor protein caspase-1 recruitment domain amino acid transporter-2 and SLC7A5 can impair the induction of helper T cell 1 and helper T cell 17 cells while weakening T cell activation.^[90,91]^ Additionally, AKG can act as a regulator of CD4(+) T cell differentiation, promoting the differentiation of naïve CD4(+) T cells into TH1 cells rather than Treg cells. Reducing extracellular glutamine levels can decrease intracellular AKG levels, favoring Treg cell differentiation.^[92]^ These findings collectively highlight that the regulatory effects of glutamine on T cell activation vary under different dosage conditions or in distinct physiological contexts. In conclusion, Gln supplementation demonstrates promise in mitigating inflammation and alleviating DCM by modulating various inflammatory signaling pathways and immune cell responses. Nevertheless, conflicting results suggest that the effects of glutamine on inflammation in DCM may be context-dependent and require further investigation.

## 6. Ferroptosis

Ferroptosis is a recently discovered regulated form of cell death that is iron-dependent and differs from apoptosis and necroptosis. It is characterized by iron overload leading to the accumulation of lipid hydroperoxides, which damage cell membranes and ultimately result in cell death.^[93,94]^ Iron overload and inactivation/deficiency of glutathione peroxidase 4 (GPX4) are the two major causes of ferroptosis.^[95,96]^ GPX4 is a selenium-containing enzyme that specifically catalyzes the conversion of toxic lipid hydroperoxides to nontoxic lipid alcohols, and its activation can abrogate the process of ferroptosis. Recent findings underscore the critical role of ferroptosis in the development of diabetic cardiomyopathy.^[97–99]^ Iron overload can exacerbate cardiovascular complications in diabetic patients through the Fenton reaction.^[100]^ Wang et al^[101]^ found that advanced glycation end-products induce ferroptosis by increasing lipid peroxide levels and decreasing the expression of ferritin and recombinant Solute Carrier Family 7 Member 11, resulting in iron overload and GSH deficiency, which ultimately leads to DCM in T2D mice. Treatment with sulforaphane can dramatically increase the levels of recombinant Solute Carrier Family 7 Member 11, ferritin, and GSH levels through the activation of the redox-sensitive transcription factor, nuclear factor erythroid 2-related factor 2 (Nrf2), thereby alleviating DCM. The essential role of ferroptosis in the pathology of DCM was also found in a diabetic rabbit model. GPX4 protein levels were significantly decreased in diabetic myocardial tissues in the rabbit model, and overexpression of Nrf2 increased the expression of GPX4, which alleviated cell injury caused by high glucose.^[102]^ Furthermore, activation of NRF2/ferroportin1 can mitigate diabetic myocardial injury by inhibiting ferroptosis.^[103]^ Notably, the hypoglycemic agent canagliflozin has shown promise in improving DCM by suppressing ferroptosis.^[104,105]^

As mentioned previously, dysfunction of GPX4 is crucial for the initiation of ferroptosis in DCM. In addition to directly inhibiting GPX4, the depletion of GSH, an essential cofactor for GPX4 function that is synthesized from Glu and cysteine, also disrupts GPX4 function, ultimately resulting in ferroptosis.^[106]^ Briefly, extracellular cystine and intracellular Glu are exchanged through System Xc at a 1:1 ratio, and then cystine is converted to cysteine, which is indispensable for the synthesis of GSH. System Xc is a chloride-dependent and sodium-independent antiporter of Cys and Glu driven by concentration gradients from extracellular Cys and intracellular Glu. Glutaminolysis, which serves as the primary supply of Glu, is crucial to ferroptosis sensitivity according to research.^[107]^ Studies have shown that down-regulation of Gln transporters SLC1A5 and solute carrier family 38 member 1 can impair the import of Gln, inhibiting ferroptosis sensitivity.^[108,109]^ In cardiac cells, silence of GLS1 can protect cells from ferroptosis in the early stage.^[110]^ Consistently, Suzuki et al^[111]^ showed that excess GLS2 favors ferroptosis by facilitating the transformation of Glu into AKG, which results in the enhancement of lipid reactive oxygen species (ROS) production, and down-regulation of GLS2 can block ferroptosis. Moreover, elevated ROS levels resulting from AKG accumulation can promote ferroptosis by enhancing lipid peroxidation and activating TP53.^[112]^ Additionally, glutaminolysis can regulate ferroptosis by modulating the activation of Nrf2. Overexpression of Gln and Glu has been shown to inhibit the Nrf2- Kelch-like ECH-associated protein 1 - Heme Oxygenase-1)/NADH Quinone Oxidoreductase 1 signaling pathway and induce ferroptosis and apoptosis.^[113]^ Jiang et al^[114]^ also demonstrated that mice hippocampal neurons cells were rescued from Glu-induced ferroptosis with Gastrodin treatment.

While a considerable body of research supports the notion that glutamine metabolism promotes ferroptosis, there is also a substantial body of literature that opposes this view. Glutamine deprivation, for example, can induce ferroptosis by reducing GSH production, which normally inhibits ROS formation, as GSH is an essential component for its synthesis.^[115,116]^ Upregulation of SLC1A5, a transporter involved in glutamine uptake, has been shown to suppress ferroptosis by enhancing GSH levels in tumor cells.^[117,118]^ These studies indicated that glutamine metabolism in ferroptosis regulation is complex, and the balance of glutamine-related pathways may play crucial roles in determining ferroptosis susceptibility in DCM.

## 7. O-GlcNacylation

O-GlcNAc is a dynamic post-translational protein modification in which the monosaccharide O-GlcNAc is attached to serine and threonine residues in proteins located in the nucleus or cytoplasm, by N-acetylglucosamine transferase, and can be removed by O-GlcNAcase (OGA).^[119–121]^ Another metabolic pathway for Gln and glucose is the hexosamine biosynthetic pathway (HBP), which modulates the generation of Uridine 5′-diphospho-N-acetylglucosamine (UDP-GlcNAc), a donor substrate for N-acetylglucosamine transferase.^[122]^ The HBP can be briefly described as follows: glucose is first phosphorylated by hexokinase to generate glucose-6-phosphate, which is then converted into fructose-6-phosphate by phosphoglucose isomerase. At this point, the metabolic pathway diverges, and the rate-limiting enzyme of the HBP, Gln fructose-6-phosphate aminotransferase 1, converts fructose-6-phosphate into glucosamine-6-phosphate. Gln and the isomerization of fructose-6-phosphate then irreversibly catalyze the conversion into glucosamine-6-phosphate and glutamate.^[123]^ Therefore, the intake, synthesis, and hydrolysis of Gln have a significant impact on the level of O-GlcNac, since it is an important substrate for O-UDP-GlcNAc. On the contrary, Petrus et al^[124]^ have reported that administration of Gln can reduce O-GlcNAc levels by inhibiting glycolysis and lowering UDP-GlcNAc in human adipocytes. These findings suggest that the regulation of O-GlcNAc levels by glutamine varies under different physiological conditions.

As a nutrient and stress sensor, O-GlcNAc is involved in the pathology of many diseases by participating in a wide range of biological processes, including transcription, signal transduction, cell cycle progression, and cell metabolism.^[122,125]^ Considerable evidence suggests that the elevation of O-GlcNAc is linked to high glucose levels in diabetes,^[126,127]^ and partly contributes to the pathogenesis and progression of DCM by causing energy deficits. Excessive O-GlcNAc has been associated with the progression of diabetes-induced cardiac fibrosis.^[128]^ Activation of OGA can significantly attenuate pathologic remodeling and heart failure.^[129,130]^ The sarco-endoplasmic reticulum calcium ATPase 2a (SERCA2a) is critical for sequestering cytosolic calcium into the sarco-endoplasmic reticulum (SR) and regulating cardiac muscle relaxation. In T2DM mice, an increase in the rate-limiting enzyme of the hexosamine biosynthetic pathway, Gln: fructose-6-phosphate aminotransferase, was detected, leading to an increase in O-GlcNAc and a decrease in SERCA2a expression. Overexpressing OGA can elevate SERCA2a expression by decreasing its O-GlcNAc level, thereby improving cardiac function in diabetic mice.^[18]^ Clark et al^[131]^ have reported that the downregulation of O-GlcNAc significantly enhances calcium transients and increases SERCA2a protein levels in myocytes exposed to high glucose. Previous study revealed that by binding to different regions in the association domain of α-kinase Anchoring Protein, SERCA2a can co-locates with Ca2+/calmodulin-dependent protein kinase II at the sarco-endoplasmic reticulum (SR) in cardiomyocytes, regulating the Ca2+/calmodulin-dependent phosphorylation of Thr-17 on phospholamban, suggesting that Ca2+/calmodulin-dependent protein kinase II may be involved in the O-GlcNAc modulation of cardiac function.^[132]^ In keeping with these results, Lu et al^[133]^ showed that diabetic hyperglycemia facilitates the O-GlcNAc of CaMKIIδ at S280, inducing the acute generation of ROS by NOX2 in cardiac myocytes. Furthermore, dysregulation of O-GlcNAc has been implicated in cardiac mitochondrial dysfunction associated with diabetes.^[134]^ In cardiomyopathy mice, a moderate increase in O-GlcNAc levels has been linked to mitochondrial dysfunction, cardiac hypertrophy, fibrosis, and diastolic dysfunction.^[135]^

Although many studies have shown that O-GlcNAc has negative effects on the heart in diabetes, there are also opposing views. For example, Kronlage et al^[136]^ confirmed that O-GlcNAc on histone deacetylase 4 at Ser-642 can inactivate pathological Ca^2+^/calmodulin-dependent protein kinase II signaling and exert a cardioprotective role in DM. HSP 70 is known for its myocardial protective effect, and studies have shown that Gln can enhance HSP 70 expression through O-GlcNAc, thereby attenuating cardiomyocyte damage.^[16,137,138]^ Although the effect of O-GlcNAc on DCM is controversial, some studies have shown that some hypoglycemic drugs can play a protective role in DCM by regulating O-GlcNAc. It has been reported that sodium-glucose cotransporter 2 inhibitor treatment can reverse the excessive O-GlcNAc in diabetic mice and prevent the development of hypertrophic cardiomyopathy.^[139]^ These studies indicate that O-GlcNAc mediated by HBP could be a therapeutic target for DCM.

## 8. Can Gln be targeted as a therapeutic approach for DCM?

Currently, specific therapeutic approaches targeting Gln for the treatment of DCM are lacking, primarily due to the complex and interconnected nature of Gln metabolism. Nonetheless, research has identified several signaling pathways and biological processes regulated by Gln metabolism that present crucial therapeutic targets for DCM. Notably, Nrf2 and NF-κB have emerged as essential targets for inhibiting inflammation and oxidative stress in DCM.^[44,140–147]^ Gln has been found to exhibit anti-inflammatory and anti-oxidative stress properties by inhibiting NF-κB and activating Nrf2.Promisingly, certain molecules and drugs have shown their potential to ameliorate DCM by targeting Nrf2 or NF-κB. For example, LCZ696, an angiotensin receptor-neprilysin inhibitor, demonstrated the ability to improve inflammation and oxidative stress in cardiomyocytes under high glucose conditions by inhibiting nuclear transfer of NF-κB and JNK/p38 mitogen-activated protein kinases phosphorylation.^[44]^ Similarly, Piceatannol, a natural hydroxylated analog of resveratrol, can alleviate inflammation and oxidative stress induced by high glucose by inhibiting NF-κB and activating Nrf2.^[148]^ Moreover, Empagliflozin, an sodium-glucose cotransporter 2 inhibitor, has shown promise in improving myocardial oxidative stress injury and cardiac fibrosis by activating Nrf2/ARE signaling.^[143]^ Additionally, Pioglitazone has demonstrated the ability to ameliorate DCM in T1DM by depressing cardiac CaMKII/NF-κB signaling and enhancing PPAR-γ expression.^[149]^ In addition to these approaches, inhibiting ferroptosis has proven effective in treating DCM, and glutaminolysis appears to contribute to ferroptosis in this context. For instance, Sulforaphane can prevent ferroptosis-associated cardiac injury in diabetic mice by activating Nrf2 mediated by AMPK.^[101]^ Another important factor in the treatment of DCM is O-GlcNAc, mediated by HBP. Vitamin D has been found to contribute to the alleviation of DCM by reducing O-GlcNAc mediated by the hexosamine pathway.^[17]^ Interestingly, exercise has been shown to significantly enhance O-GlcNAc in the diabetic heart and subsequently reduce the level of the mSin3A/HDAC1/2 transcription factor complex, suggesting the complex function of O-GlcNAc in diabetic cardiac health.^[150]^ In conclusion, targeting Gln-regulated signaling pathways and biological processes holds promise for effectively improving DCM. While directly targeting Gln metabolism remains a potential avenue for more comprehensive and effective therapeutic effects, current research supports the importance of focusing on specific pathways influenced by Gln in the treatment of DCM. Advancing our understanding of these pathways may pave the way for more targeted and effective therapeutic interventions in the future.

## 9. Conclusion

The regulation of Gln metabolism and its downstream signaling pathways shows great promise as a therapeutic strategy for DCM, as Gln and its metabolites have been shown to prevent oxidative stress injury, inflammation, ferroptosis, and apoptosis in DCM by regulating multiple signaling pathways. However, the suitability of Gln as a therapeutic target for preventing and treating DCM presents several unanswered questions. Firstly, the predominant body of research into the role of Gln metabolism in DCM progression is grounded in basic research, with limited clinical investigations. Further comprehensive studies are imperative to conclusively ascertain the potential benefits of Gln supplementation for ameliorating DCM within clinical contexts. Secondly, Gln metabolism encompasses diverse downstream mechanisms, rendering the precise regulation of Gln a dual-edged sword. While several studies suggest that Gln may enhance DCM outcomes by mitigating oxidative stress, inflammation, iron-induced cell death, or reducing O-GlcNAc levels, other research has indicated that, under different physiological conditions or in cases of excessive Gln metabolism, it may stimulate inflammatory responses by activating T cells or elevate ROS levels, leading to cellular iron-induced death. Moreover, Gln, serving as a critical substrate for O-GlcNAc modification, may potentially elevate O-GlcNAc modification levels, thereby potentially accelerating the progression of DCM. Consequently, the feasibility of targeting Gln metabolism necessitates further elucidation. Lastly, given the energy demands of Gln metabolism and the variances in downstream regulatory mechanisms across different cell types, especially between myocardial cells and other cell types, selective regulation of Gln metabolism in myocardial cells is imperative to mitigate potential adverse off-target effects. Additionally, comprehending the intricate regulatory mechanisms of Gln metabolism across diverse types of diabetes-related cardiomyopathies warrants attention in future research endeavors. Addressing these multifaceted issues promises to foster the development of novel therapeutic agents with the goal of reducing the incidence and mortality associated with diabetic cardiovascular complications.

## Author contributions

**Conceptualization:** Yiying Zhang.

**Data curation:** Yiying Zhang.

**Writing – original draft:** Yiying Zhang.

**Writing – review & editing:** Yiying Zhang.
